# Viable Triploid Honey Bees (*Apis mellifera capensis*) Are Reliably Produced in the Progeny of CO_2_ Narcotised Queens

**DOI:** 10.1534/g3.118.200614

**Published:** 2018-08-25

**Authors:** Benjamin P. Oldroyd, Sarah E. Aamidor, Gabriele Buchmann, Michael H. Allsopp, Emily J. Remnant, Fan F. Kao, Rebecca J. Reid, Madeleine Beekman

**Affiliations:** * Behaviour and Genetics of Social Insects Laboratory, Macleay Building A12, University of Sydney, NSW 2006, Australia; † Honeybee Research Section, ARC-Plant Protection Research Institute, Private Bag X9017, Stellenbosch 7599, South Africa; ‡ Sydney Cytometry, University of Sydney and Centenary Institute, NSW 2006, Australia

**Keywords:** Thelytokous parthenogenesis, haplo-diploidy, central fusion, triploid

## Abstract

The haplodiploid system of sex determination of Hymenoptera acts as an exaptation for species to evolve novel forms of asexual reproduction including thelytoky (clonal offspring of the mother). During normal reproduction in Hymenoptera, three of the four products of meiosis that are present in newly-laid eggs are lost as polar bodies, while the remaining pronucleus either develops as a haploid male or fuses with a sperm nucleus to produce a diploid zygote. In contrast, in thelytokous reproduction, which is uncommon but taxonomically widespread, two of the four products of meiosis fuse, as if one acted as a sperm. Queenless workers of *Apis mellifera capensis*, a subspecies of honey bee from South Africa, routinely reproduce thelytokously. Unmated *A. m. capensis* queens can also be induced to lay thelytokously by narcosis with carbon dioxide, but mated queens are never thelytokous. We artificially inseminated *A. m. capensis* queens using CO_2_ narcosis. Up to 1/3 of offspring workers carried two maternal alleles and an allele of one father whereas no three-allele progeny were seen in control queens of the arrhenotokous (unfertilized eggs result in males) subspecies *A. m. scutellata*. Flow cytometry of three-allele individuals revealed that they were triploid and arose from the fertilization of a thelytokous fusion nucleus. We then reared six queens from a narcotized *A. m. capensis* queen and determined the ploidy of the offspring queens based on microsatellites. One of the five daughters was triploid. Following artificial insemination, this queen produced unfertilized thelytokous diploid eggs at high frequency, and unfertilized triploid eggs at much lower frequency. If fertilized, thelytokous diploid eggs were non-viable, even though triploidy in itself does not impede normal development. In contrast, when the rarer triploid eggs were fertilized, a proportion developed into viable tetraploids. Our study highlights the extraordinary developmental flexibility of haplo-diploid systems.

In Hymenoptera (ants, wasps and bees) males are haploid and females are diploid (White 1973). Often, the switch that determines sex is a single locus, the *complementary sex determiner* (*csd*) (Cook and Crozier 1995; Beye *et al.* 2003; Heimpel and de Boer 2008). When an individual is heterozygous at *csd*, it develops as a female. Eggs that are hemizygous or homozygous at *csd* develop as a male, but homozygotes develop into diploid males, which, in honeybees, are eaten by workers at the first larval instar (Woyke 1963).

Haplodiploidy acts as an exaptation (sensu Gould and Vrba 1982) for unusual modes of reproduction. In particular, there is no impediment to an unfertilized egg developing into an adult (Heimpel and de Boer 2008). That is, given the right conditions a haploid pro-nucleus can divide mitotically and produce an embryo or part of an embryo. Unlike mammals and most other insects, fertilization is not necessary for development (Sasaki and Obara 2002).

The possibility of egg development without the need of fertilization gives rise to some interesting anomalies (Schwander and Oldroyd 2016). First, because the eggs of some or most haplodiploids are polyspermic (two or more sperm enter each egg, Baer *et al.* 2016), it is possible to have fusion between a maternal pro-nucleus and a sperm nucleus to generate a normal diploid zygote, while one or more of the additional sperm also start dividing producing haploid male tissue. These individuals, known as gynandromorphs, are mosaics of bi-parental female tissue and paternally-derived haploid male tissue (Rothenbuhler *et al.* 1952; Drescher and Rothenbuhler 1963; Rothenbuhler 1954). Such individuals are expected to carry one maternal and two or more paternal alleles at some loci.

Second, if a sperm cell fertilizes an anucleate egg, a sperm nucleus can potentially give rise to a clone of the sperm donor by ‘androgenesis’. Androgenesis is a well-established phenomenon in the honeybee *Apis mellifera* (Koeniger *et al.* 1989) and three ant species (Fournier *et al.* 2005; Kobayashi *et al.* 2008; Pearcy *et al.* 2004). In some ants a combination of androgenesis and thelytoky leads to the extraordinary situation in which queens clone themselves to produce daughter queens, and males clone themselves to produce sons (Fournier *et al.* 2005; Foucaud *et al.* 2007).

Third, two sperm can fuse in an egg to produce diploid female tissue (Laidlaw and Tucker 1964). Thus far we only have reports of mosaics: mixtures of tissues derived from the fusion of two sperm and the fusion of a maternal pronucleus and a sperm nucleus. However, it is plausible that females that are entirely or mostly derived from the fusion of two sperm are possible.

Finally, embryos can be derived from the fusion of two maternal pronuclei, giving rise to females by thelytokous parthenogenesis. Whereas the unusual forms of egg development discussed above are rare, thelytoky is widespread in Hymenoptera (Rabeling and Kronauer 2013).

Honey bee eggs are laid arrested in anaphase I (Snodgrass 1956). Following oviposition, the primary oocyte completes meiosis I to produce the two secondary oocytes. These then undergo second division meiosis resulting in four maternal pronuclei (Verma and Ruttner 1983; Cole-Clark *et al.* 2017). In most subspecies three of the four maternal pronuclei degenerate (Snodgrass 1956). If the egg has been fertilized the remaining pronucleus will fuse with a sperm nucleus to produce a female. If the egg has not been fertilized the remaining pronucleus will divide mitotically and produce a male via arrhenotokous parthenogenesis.

In the southern provinces of South Africa there exists a unique honey bee subspecies which is thelytokous (reviewed in Beekman *et al.* 2008). In this subspecies, *A. m. capensis*, (hereafter Capensis) queenless workers lay eggs that mostly result in females via thelytokous parthenogenesis, whereas in all other honey bee species and subspecies such as the African bee *A. m. scutellata* (hereafter Scutellata), unmated workers produce male offspring via arrhenotokous parthenogenesis (Goudie and Oldroyd 2014) (but see Remnant *et al.* 2014; Tucker 1958; Mackensen 1943).

Here we examine the progeny of *A. m. capensis* queens that had simply been instrumentally inseminated; a process that requires CO_2_ narcosis. We discovered up to 1/3 of eggs and adult workers carried two maternal alleles and one paternal allele. We determine the nature of these individuals (triploid or mosaics of haploid and diploid tissue) using a combination of molecular markers and flow cytometry. We also report on the viability and ploidy of the progeny of a single triploid queen.

## Materials And Methods

### Experiment 1. CO_2_ narcosis induces three-allele progeny

**Figure 1:**
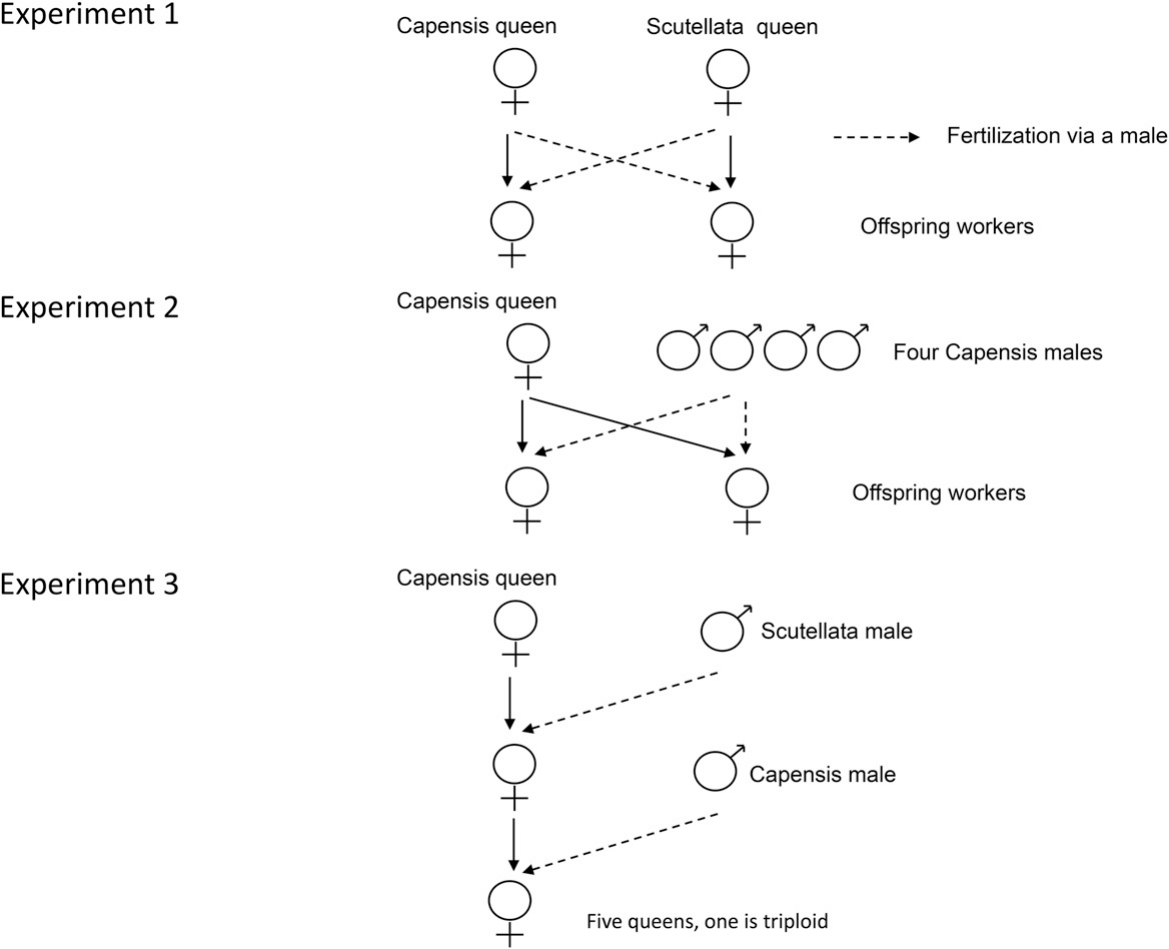
Crossing designs. Experiment 1. Four *A. m. capensis* and four *A. m. scutellata* queens were crossed in reciprocal. Pre-emergent worker pupae were collected and genotyped. Experiment 2. Two *A. m. capensis* queens were inseminated with semen from four unrelated *A. m. capensis* males. Offspring workers were genotyped and their ploidy levels determined by flow cytomentry. Experiment 3. An *A. m. capensis* queen was inseminated with the semen of a single *A. m. scutellata* male. Five daughter queens were reared from this queen, and each was inseminated with the semen of a single *A. m. capensis* male. One of the five queens was triploid.

In Stellenbosch South Africa, we inseminated four Capensis virgin queens each with the semen of a single Scutellata male and four Scutellata queens, each with the semen of a single Capensis male (Figure 1). Virgin queens were introduced to small colonies and prevented from leaving on mating flights by grids placed on the front of the hives. We used standard insemination procedures that involved relatively brief narcosis during insemination (2-3 min), and a 10 min narcosis the day after insemination to induce oviposition (Harbo 1986). We retained the males and the wing clippings of the queens for later genotyping, and harvested pupae from worker cells for genotyping. Samples were stored in 95% ethanol at -20C. We then genotyped the parents and 12-25 mature pupal progeny from worker cells at microsatellite loci A8, A14, A79, A88, A113, B124, A107, Ap43 (Solignac *et al.* 2003) and HB-The-03 (Shaibi *et al.* 2008).

### Experiment 2. Are the three-allele individuals triploids or mosaics?

During Experiment 1 we found that a significant proportion of offspring of the four Capensis queens carried three alleles at multiple loci. To clarify whether these offspring were triploids or mosaics we required frozen bees that were suitable for flow cytometry. We therefore inseminated two new Capensis queens, each with the semen of four Capensis drones unrelated to the queen or each other as above. We retained the queens’ wing clippings and the drones in 95% alcohol for genotyping. Pre-emergent workers (PEW, n = 200 per colony) were collected on to dry ice nine weeks post-insemination, thereby ensuring that all progeny originated from the inseminated queen.

We genotyped queens, drones and worker offspring pupae using microsatellite loci A8, A29, A79, A88, A113, B124 and Ap43 (Solignac *et al.* 2003), determining the number of alleles at each locus and the parental origin of each allele. From this analysis we randomly selected four workers carrying two alleles and four workers carrying three alleles at at least four of the seven loci from each colony. In addition, we sampled one diploid worker and one haploid drone from Sydney Australia, and three haploid *A. m. capensis* males to provide reference specimens for ploidy.

Flow cytometry was then used to determine the ploidy of the selected PEW. Because some honey bee tissues are known to undergo autopolyploidization, we sampled tissue from both brain (which does not undergo autopolyploidization) and thorax (which does, at least in males) (Aron *et al.* 2005).

Dissected tissue was suspended in 200 µl Galbraith Buffer (45 mM magnesium chloride, 30 mM sodium citrate, 20 mM 4-morpho-G line propane sulfonate and Triton X-100 (1 mg/mL)) and homogenized to release the nuclei with a Tenbreck homogenizer using six gentle strokes. The tissue was then filtered through 10 µm nylon mesh (Rangel *et al.* 2015). To stain the nuclei, 1 µl of DAPI staining solution (10 mg/ml) was added to each sample and incubated for 15 min at 4° in the dark.

The DNA content of each nucleus was measured by its relative fluorescence using a 5-laser Becton Dickinson LSR-II flow cytometer (BD Bioscience, San Jose, CA). The DNA-bound DAPI was excited with either a 20 mW 355 nm ultraviolet (UV) laser or a 50 mW 405 nm violet laser. The fluorescence was detected using PMT detectors equipped with a 450/50 bandpass filter. The same fluorescent detector gain settings were used for all samples. The gates for the identification of nuclei and the analysis of fluorescence intensities were consistent across all samples.

The brain tissue of the Australian drone and worker were used to identify C1 (haploid) and C2 (diploid) fluorescent peaks respectively. Peaks representing C1, C2, and C4 (tetraploid, most likely dividing cells) in Capensis drones and Capensis two-allele and three-allele workers, were then inferred from these standards. Frequency histograms of the number of cells of each level of ploidy where produced using BD FACSDiva software version 8.0.1 (BD Bioscience, San Jose, CA).

### Experiment 3. A viable triploid queen and her progeny

In our final experiment we inseminated a Capensis queen with semen from a single Scutellata male using CO_2_ narcosis as above. We then reared six super-sister Capensis x Scutellata queens from this individual and inseminated each with the semen of a single (but different) *A. m. capensis* male (Figure 1). It is important to note that these queens were daughters of a CO_2_-narcotised Capensis queen, even though they themselves were only 50% Capensis (Figure 1). We retained a wing clipping of each queen. We extracted DNA from the wing clippings using Chelex (Walsh *et al.* 1991) and genotyped the extract at six microsatellite loci (A14, A29, A88, A107, A113, B124, Estoup *et al.* 1993; Solignac *et al.* 2003). One of the six queens showed three alleles at four of the six microsatellite loci, indicating that she was triploid (Table S1). The other five queens were diploid.

**Figure 2:**
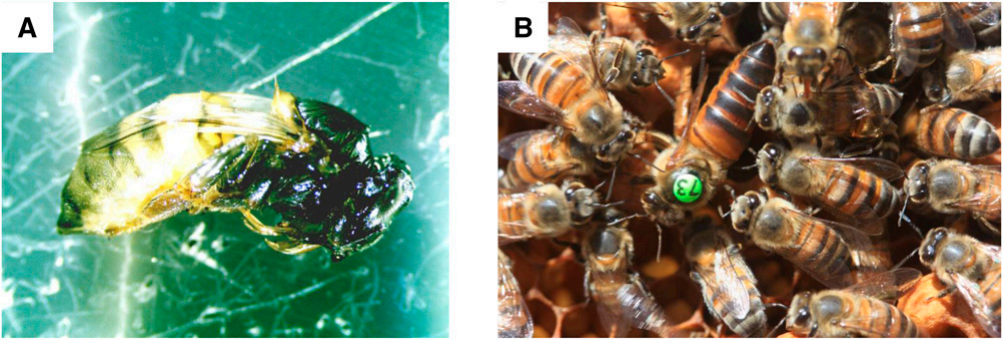
A. Pre-emergent worker with three alleles at multiple loci from an *A. m. capensis* queen x *A. m. scutellata* male cross. The female is a phenotypically normal worker, with no evidence of male tissue. B. A triploid honey bee queen (with numbered identifying tag green 73). The queen was phenotypically normal, but very few of her eggs were viable.

The triploid queen, AI 109, was phenotypically normal (Figure 2) and maintained 2-3 brood combs of eggs throughout her life from November 2017 to January 2018. During this period we supported her colony by adding frames of emerging brood from *A. m. scutellata* colonies on a monthly basis. We marked the added frames, and did not sample brood items from them for at least 22 days after addition of the donor brood. By this time all donor brood would have emerged from their cells.

#### Ploidy of the triploid queen’s brood:

On two occasions, we collected all brood items beyond egg stage, and about 100 eggs, from AI 109’s colony. We genotyped progeny based on the microsatellite loci listed above, and determined whether they were progeny of AI 109 those of the unrelated workers that comprised her colony, and the genotypic ploidy of each individual.

#### Egg viability:

To quantify the proportion of eggs that hatched we cut small pieces of brood comb containing c.a. 100 eggs from AI 109’s colony and placed them in a zip-lock bag containing a moist paper towel, and placed the bag in an in an incubator at 34.5C. As a control, we cut similar pieces of comb from the brood combs of three diploid queens that were super-sisters of the triploid queen.

### Data availability statement

File S1 lists the microsatellite genotypes of pupae from reciprocal crosses between Capensis and Scutellata queens and drones (Experiment 1). File S2 lists the microsatellite genotypes of the four two-allele and four three-allele individuals used for flow cytometry (Experiment 2). File S3 provides the genotypes of the six daughter queens of a Capensis queen x Scutellata male cross (Experiment 3). File S4 provides the microsatellite genotypes of the triploid queen and her brood. Supplemental material available at Figshare: https://doi.org/10.25387/g3.6873998.

## Results

### Experiment 1. CO_2_ narcosis induces triploidy in the progeny of *A. m. capensis* queens

All four Scutellata queens inseminated with a Capensis male produced phenotypic females that had normal diploid genotypes at each locus, or an expected homozygote where the queen and the inseminating male carried the same allele (Table 1). All four of the Capensis queens inseminated with an *A. m. scutellata* male also produced normal diploid offspring, but also some phenotypically normal female progeny (Figure 2), that carried three alleles, two maternal and one paternal, at multiple loci (Table 1). On average, 14% (range 5–33%) of offspring per colony carried three alleles at multiple loci.

**Table 1:** Number of mature pupal female progeny sampled per colony (a-h) with a maximum of two alleles per locus and a maximum of three alleles per locus in reciprocal crosses between *Apis mellifera capensis* and *Apis mellifera scutellata*

	Scutellata queen x Capensis male				Capensis queen x Scutellata male			
Maximum number of alleles per locus	a	b	c	d	e	f	g	h
2	12	20	18	25	17	18	12	17
3	0	0	0	0	1	2	6	1

We verified the three-allele genotypes with a second PCR of the hind leg and the front leg. The hind leg and the front leg had identical genotypes in all cases suggesting that the individuals were either triploid or a homogeneous mosaic of two or more diploid cell lines that shared the same maternal allele. There was no evidence of androgenesis or thelytoky in any progeny.

### Experiment 2. Are three-allele individuals triploids or mosaics?

The two queens each produced phenotypically normal female progeny with 5% and 8% (n = 200 per colony) of daughters carrying three alleles at multiple loci respectively. Genotyping revealed that all these individuals carried two maternally-derived alleles and one paternal allele at each of the three-allele loci (Table S2).

**Figure 3:**
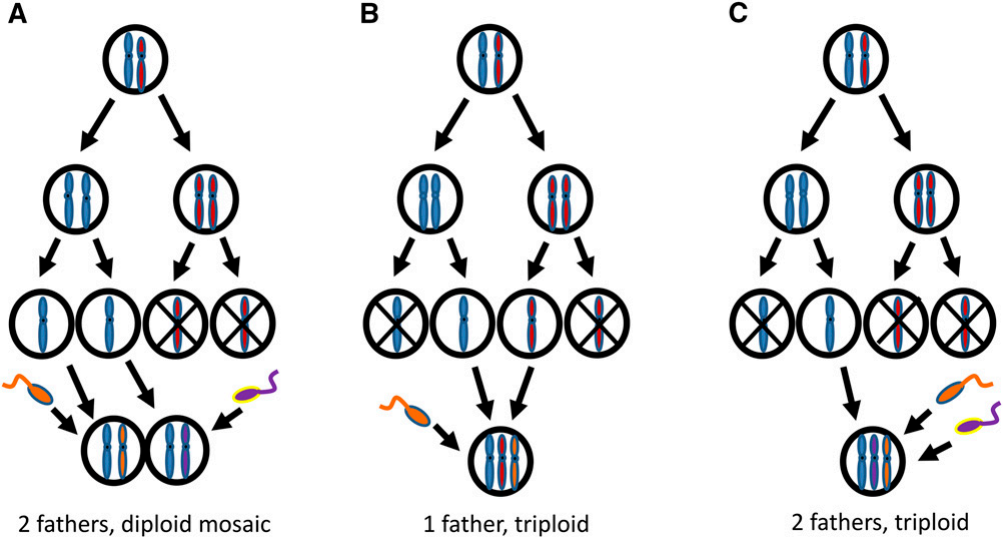
Plausible mechanisms for the creation of individuals with three alleles at multiple loci. (a) The two terminal pronuclei fuse with different sperm pronuclei creating a diploid mosaic. Other variations are possible including independent fusions of the two central pronuclei with identical sperm produced by a single father. (b) The two central pronuclei fuse with a single sperm pronucleus to produce a triploid. (c) A single maternal nucleus fuses with two sperm of different fathers.

We suggest that there are three plausible scenarios that could give rise to the three-allele individuals identified based on microsatellite markers (Figure 3). (a) A mosaic diploid individual could arise from two maternal pronuclei each separately fusing with a different sperm nucleus, giving rise to an individual that is a mosaic of diploid cells descended from two or more zygotes. In this case the flow cytometry would reveal C2 nuclei and the microsatellites two maternal and one or more paternal alleles. (b) A triploid individual might arise from the fusion of sperm with two maternal pronuclei. In this case flow cytometry would show that the majority of nuclei were C3 and the microsatellite analysis would show two maternal alleles and one or more paternal alleles. (c) Two sperm nuclei enter an egg and fuse with a single maternal pronucleus. Under this hypothesis we predict that cytometry would show the individual as C3 and the microsatellite genotyping would show one maternal and two paternal alleles.

**Figure 4:**
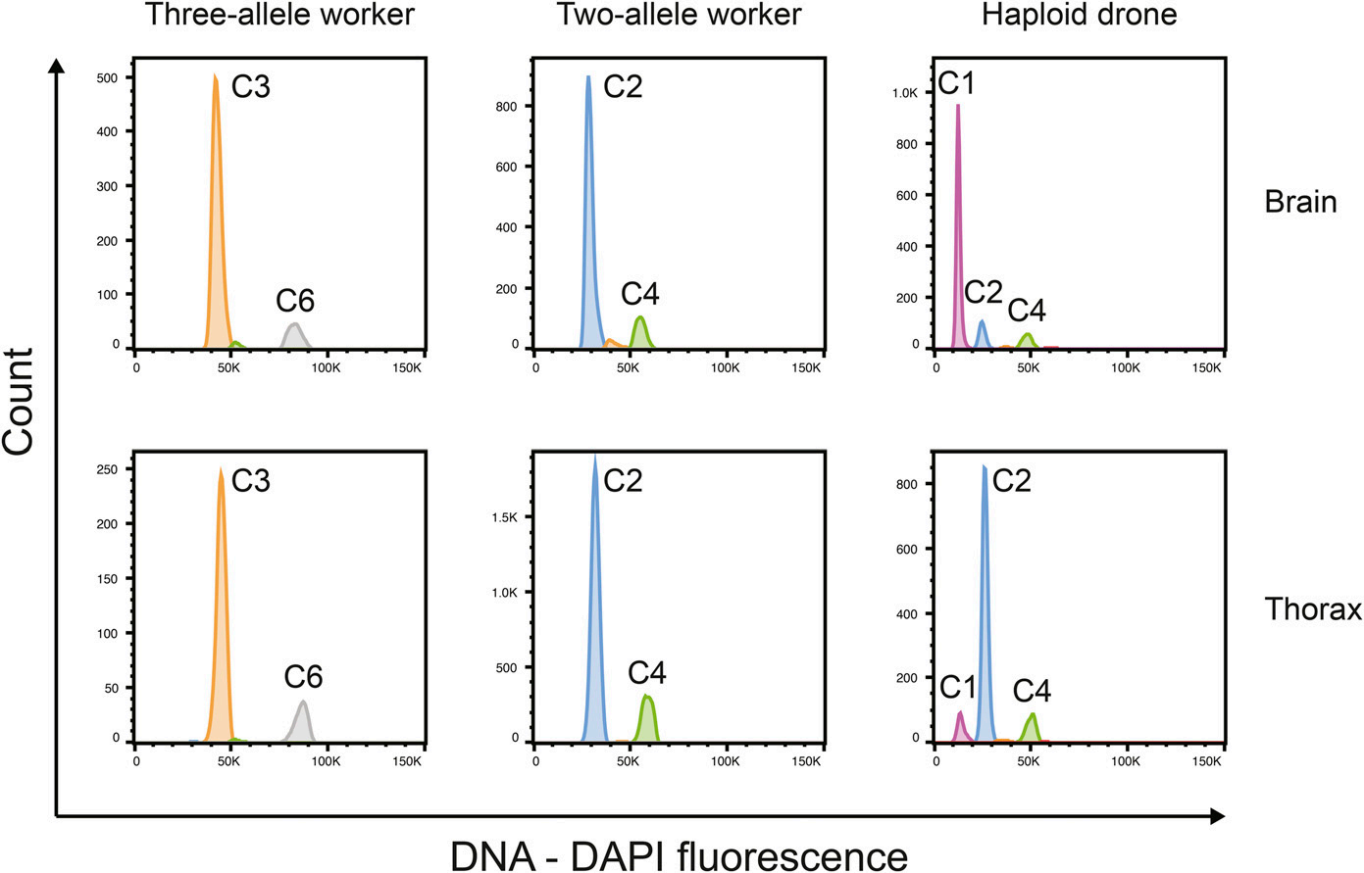
Flow cytometry histograms showing the nuclei count (Y axes) at different fluorescence levels (X axes). Each peak represents the ploidy level in the tissue sample in brain and thorax for workers with two or three alleles at multiple loci. The two right hand panels are for the single haploid *A. m. capensis* drone that was used to calibrate the flow cytometry results. This in turn was calibrated against an Australian worker and drone (data not shown). In the male, the brain tissue is haploid and the thoracic tissue is diploid as a result of autopolyploidization (Aron *et al.* 2005).

In order to verify whether our three-allele individuals where true triploids or diploid mosaics with two cell lines, we determined the ploidy of four 2-allele and four 3-allele individuals per colony by flow cytometry (Figure 4). To correct for variation in the number of cells per sample we converted counts of 2n and 3n cells to the proportion of the total cell count. Data were then analyzed using two generalized linear models, as implemented in SPSS, of the effect of tissue (brain *vs.* thorax) and allele number (2 alleles *vs.* 3 alleles) on the frequency of cells classified as 3n or 2n with a normal link function. We nested the effect of individual bee within allele number.

**Figure 5:**
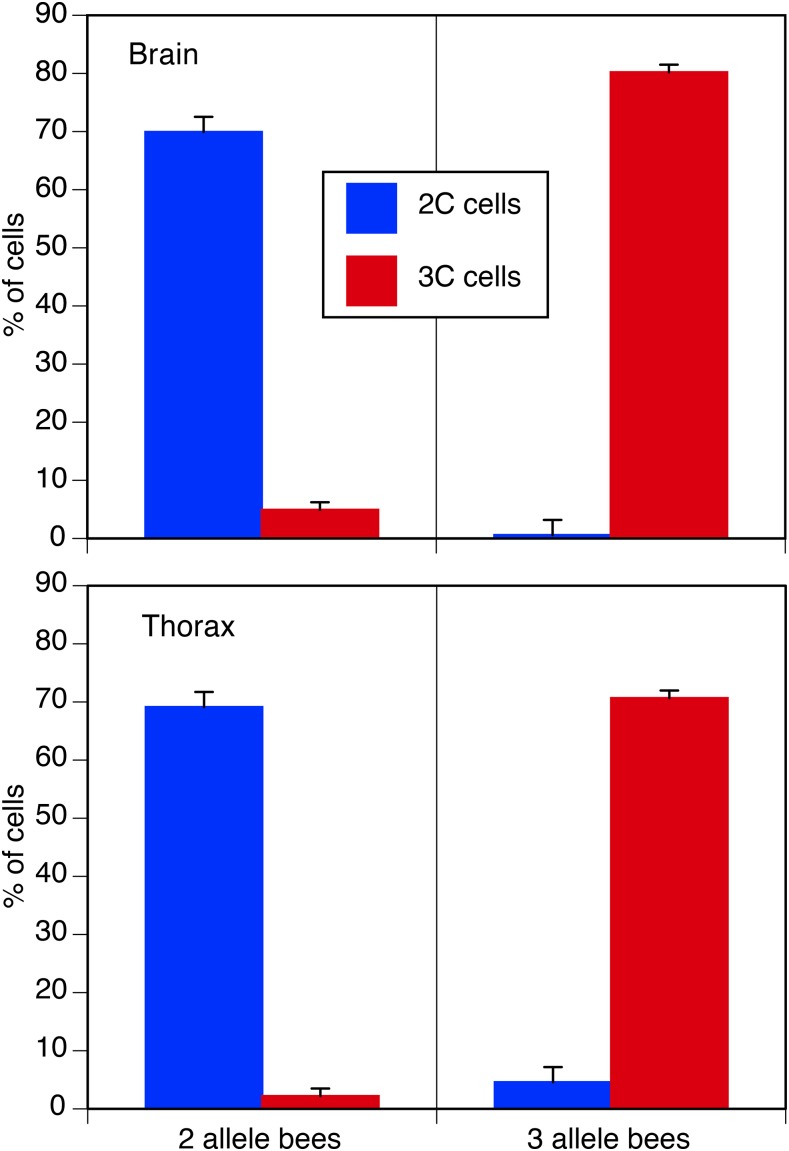
Mean proportion of nuclei (%) measured as being diploid or triploid in two tissue types (brain and thorax) by flow cytometry for individuals showing two or three alleles at multiple loci. Error bars are standard error of the mean. n = eight workers for three alleles and eight workers for two alleles.

There was a highly significant difference (*P* < 0.001) in the ploidy of cells extracted from bees with two alleles per locus compared to bees with three alleles per locus (Table 2). The vast majority of cells from three-allele individuals were triploid, whereas cells from two-allele individuals were diploid (Figure 5). In combination with the genotyping results, this is strong evidence that the three-allele individuals were triploids formed by the fusion of the two central maternal pronuclei and one sperm nucleus (hypothesis b in Figure 3).

**Table 2:** Results of two generalized linear models for the effect of allele number (2 or 3 per locus) on the proportion of cells per bee (n = 16 bees per allele number) assessed as being diploid (C2) or triploid (C3) based on flow cytometry. The effect of tissue (brain *vs.* thorax) is included in the models, with replicate bee nested within allele number

		Proportion of cells measured as diploid (C2) or triploid (C3)			
		C2		C3	
Source	d.f	Wald χ^2^	*P*	Wald χ^2^	*P*
Tissue	1	0.375	0.541	21.76	< 0.001
Allele number	1	657.492	< 0.001	2989.504	< 0.001
Bee(allele number)	14	23.8	0.048	53.775	< 0.001

Examination of the genotypes revealed no thelytokous progeny (*i.e.*, two maternal alleles and no paternal alleles). For each triploid individual only one paternal allele was detected, providing additional evidence that these individuals arose by the fusion of the two central maternal pronuclei with a sperm.

There is no evidence that any particular male’s sperm was more likely to form triploid embryos. A cross tabulation of paternity and ploidy showed no association (Queen 1, χ^2^ = 2.253, df = 3, *P* = 0.522; queen 2 χ^2^ = 5.301, df = 3, *P* = 0.151).

### Experiment 3. A viable triploid queen and her progeny

#### Ploidy of brood items:

In worker cells the majority of eggs were triploid and fertilized (Table 3, see Supplementary Table S3 for raw genotyping data). In drone cells the majority of eggs were diploid and unfertilized, but all pupae in drone cells were worker laid, suggesting that unfertilized diploid eggs laid in drone cells were non-viable.

**Table 3:** Ploidy of brood items produced by a triploid *A. m. capensis* queen

Developmental stage	Origin	Ploidy	Count	Total
Pupae	Worker cells	Tetraploid fertilized	16	
		Triploid unfertilized	3	
		Triploid fertilized	0	
		Diploid fertilized	0	
		Diploid unfertilized	0	
		Haploid	0	19
Eggs	Worker cells	Tetraploid fertilized	0	
		Triploid unfertilized	0	
		Triploid fertilized	127	
		Diploid fertilized	29	
		Diploid unfertilized	30	
		Haploid	0	186
Pupae	Drone cells	All worker laid		
Eggs	Drone cells	Tetraploid fertilized	0	
		Triploid unfertilized	10	
		Triploid fertilized	1	
		Diploid fertilized	1	
		Haploid	0	
		Diploid unfertilized	36	48

#### Egg viability:

Although the triploid queen was phenotypically normal (Figure 2) and estimated to have laid hundreds of eggs per day, like a normal queen, few of the eggs hatched. Only one in 200-300 brood cells contained a brood item beyond egg stage. None of AI 109’s 73 eggs laid in drone cells or 144 eggs laid in worker cells hatched within 72 hr in our incubator study, whereas 93.3% (range 90.0–97.5%) of the 468 diploid queen’s eggs laid in worker cells by three super-sister queens hatched within 72 hr (Table 4). This showed that AI 109 had very low egg viability, even though we were able to harvest a small number of larvae and pupae from drone and worker cells.

**Table 4:** Egg viability from a triploid queen honey bee

Queen number	Ploidy based on microsatellites	Source of eggs	Number of eggs scored	Number of eggs that hatched	Egg viability (%)
AI 109	Triploid	Drone cells	73	0	0
		Worker cells	144	3	2.1
AI 106	Diploid	Worker cells	80	78	97.5
AI 107	Diploid	Worker cells	180	162	90.0
AI 108	Diploid	Worker cells	208	193	92.7

## Discussion

This study has shown that triploid queens and workers can be reliably produced (5–33%) in the progeny of Capensis queens narcotized with CO_2_ during standard insemination procedures. The genotype of the inseminating male is irrelevant, since Capensis queens inseminated with both Capensis semen (Experiment 2) and Scutellata semen (Experiments 1 and 3) all produced triploid offspring. Triploid females arise following fertilization of a diploid nucleus that formed as a result of the thelytokous fusion of two maternal pronuclei. Although we did not study the behavior of triploid workers, the triploid queen was fully viable and produced thousands of eggs. A small proportion of her eggs were viable.

We suggest that the triploid individuals we produced arose as an interaction between the thelytokous Capensis strain and CO_2_ narcosis. Because Capensis are thelytokous the maternal pronuclei are receptive to fusion and fertilization rather than degeneration (as happens in other subspecies). Whether the sperm nucleus fuses with an already-formed thelytokous nucleus or whether the three nuclei fuse simultaneously is unclear. Our study emphasizes the extraordinarily broad range of effects of CO_2_ narcosis on honey bee behavior (Ribbands 1950; Simpson 1954; Skowronek 1976), physiology (Harris *et al.* 1996), reproduction (Koywiwattrakul *et al.* 2005), gene expression (Niño *et al.* 2011; Brito *et al.* 2010; Manfredini *et al.* 2015) and embryogenesis (this study, Oldroyd *et al.* 2008; Crewe and Allsopp 1994).

It is unlikely that triploidy is a common phenomenon in the wild *A. m. capensis* population. Triploids have not been reported in any large-scale genotyping studies of sexually-produced Capensis workers from naturally-mated queens (*e.g.*, Beekman *et al.* 2009; Moritz *et al.* 2011; Holmes *et al.* 2010; Goudie *et al.* 2012; Beekman *et al.* 2011). Triploids only seem to arise after CO_2_ narcosis, which is known to induce thelytoky in virgin Capensis queens (Crewe and Allsopp 1994; Oldroyd *et al.* 2008). We note that in Hymenoptera, triploid females can also arise as a consequence of a diploid male mating with a normal diploid female in inbred populations (Harpur *et al.* 2013; Leibert *et al.* 2004; Krieger *et al.* 1999; Ayabe *et al.* 2004; Darvill *et al.* 2012; Chaud-Netto 1980). However in honey bees, diploid males are never found in nature because diploid male larva are removed by nurse bees (Woyke 1963). Therefore this is the first report of wide-spread triploidy in outbred honey bees.

**Figure 6:**
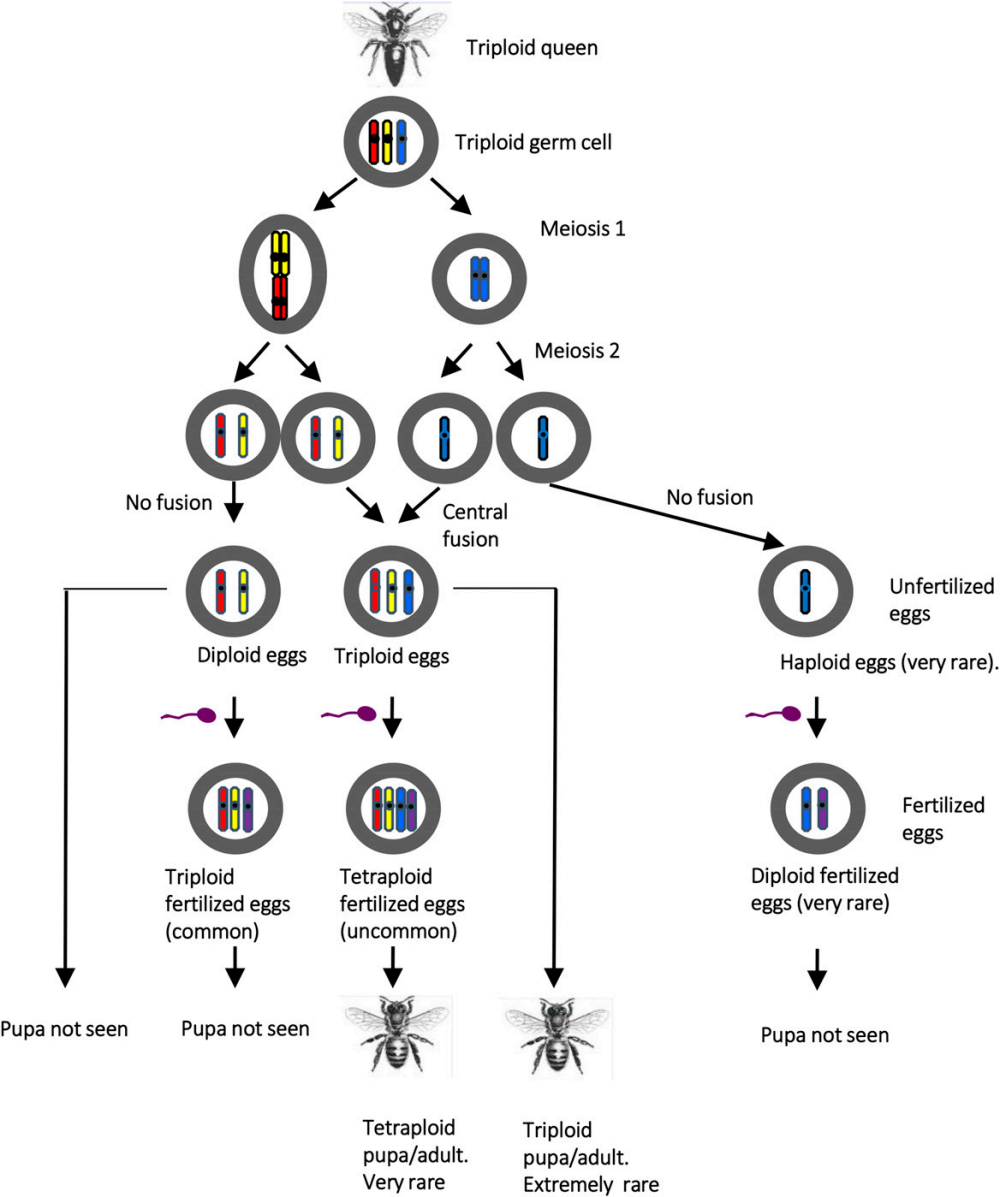
A model explaining the genetic origin of brood items observed in the progeny of a triploid honey bee queen. The model depicts the meiotic events of a single chromosome and its homologs. A triploid germ cell in the queen undergoes meiosis I. Meiosis II occurs after the egg is laid resulting in two diploid and two haploid pronuceli. A proportion of the central pronuceli fuse to produce a triploid fusion nucleus, which is usually fertilized if laid in a worker cell and not fertilized if laid in a drone cell.

In Figure 6 we propose a model describing the possible cytogenetic origins of the progeny of triploid queen. In meiosis I, we suggest that the triploid germ cell undergoes chromosome duplication and cell division to produce two daughter cells with duplicated chromosomes, one tetraploid, one diploid. The two cells of meiosis I then undergo a reductional division in meiosis II to produce two haploid and two diploid daughter cells (Figure 6). The two diploid cells carry non-sister chromosomes, though there may be some exchange of genetic material between the two because of recombination in meiosis I meaning that some loci that were heterozygous in the mother will be homozygous in the offspring (Goudie and Oldroyd 2014).

Below we refer to unfertilized eggs as oocytes and fertilized eggs as eggs. The majority of eggs/oocytes in worker cells were triploid and fertilized. We propose that the triploid eggs arose when a diploid oocyte was fertilized (Figure 6). However, no triploid individuals carrying paternal alleles were identified in pupae (Table 2), indicating that triploid eggs were inviable.

A few diploid eggs were found in worker cells. These probably arose when a haploid oocyte was fertilized. However, no diploid fertilized pupae were found. This is surprising because there is no reason to suspect that a diploid fertilized embryo would not be fully viable and result in a normal worker. Our failure to identify such pupae may be because diploid eggs were rare (Table 4), rather than a lack of viability. Curiously, if our model is correct, we would expect approximately equal numbers of diploid and haploid oocytes to be produced by the triploid queen (Figure 6). Therefore the much higher frequency of diploid oocytes than haploid oocytes is unexplained by our model. Potentially some diploid oocytes arose from a thelytokous fusion of haploid maternal pronuclei, rather than a diploid pronucleus.

In pupae from worker cells, most individuals were tetraploid, apparently resulting from the fertilization of a triploid oocyte (Figure 6). Tetraploid eggs were not found in worker cells, suggesting that they are rare but viable. Supporting this conjecture, triploid oocytes were the most common egg in drone cells, suggesting that triploid oocytes are produced at low frequency and can result in a viable tetraploid when laid in a worker cell and fertilized.

Most eggs produced by AI 109 were not viable. In some instances this is surprising. Haploid eggs laid in drone cells should develop normally into adult drones, but none were seen. Similarly, haploid oocytes that are then fertilized should produce viable workers, but none were seen. Our study shows that triploid female honey bees are fully viable. Why, then, were the majority of triploid fertilized eggs (the most common class of eggs in worker cells) non-viable? One possibility is that errors in chromosome disjunction during meiosis did not lead to a neat segregation of haploid and diploid female gametes as suggested in Figure 6, but to eggs with varying numbers of chromosomes, and perhaps missing chromosomes. Such eggs may be incapable of normal development. Only the occasional egg may have had a complete, balanced set of chromosomes, sufficient for proper development. The genotypes of eggs from worker cells are compatible with this hypothesis. Eggs with three or four alleles at particular loci did not show this pattern across all loci, suggesting variation in chromosome number. Nonetheless, such a pattern is also compatible with a loss of heterozygosity due to recombination at meiosis I (Goudie and Oldroyd 2014). That is an egg may have a complete haploid or diploid set of chromosomes, but two, or three chromosomes may share the same microsatellite allele, erroneously suggesting haploidy.

Alternatively, the low egg viability observed here may be a consequence of the sex determination system. *Csd* encodes an *SR*-type protein. When *csd* is heterozygous it produces a functional product that initiates development of a female phenotype by activating *AmDSX*. When homozygous or hemizygous it produces a non-functional product that allows male development by default (Beye *et al.* 2003). This suggests that if any two *csd* alleles present in a diploid, triploid, or tetraploid embryo are different, then the female development pathway should be switched on via the production of a functional csd protein. This implies that most fertilized eggs should be viable because they are expected to carry different *csd* alleles. Therefore the non-viability of most triploid and diploid eggs is most likely not a consequence of sex determination, and we therefore suggest that errors in chromosomal disjunction during the formation of eggs is the more likely explanation of low egg viability in the triploid queen.

The reliable production of large numbers of triploid workers by CO_2_ narcosis of Capensis queens provides new opportunities for studying development and sex determination mechanisms in bees. When non-Capensis honey bee queens are inseminated by related males, individuals that are diploid but homozygous at *csd* are produced among progeny. Such individuals are phenotypic males, but are removed by nurse workers at the first larval instar (Beye *et al.* 2003; Woyke 1963). An interesting experiment would be to narcotize a Capensis queen and then inseminate her with a brother. Such a queen should produce some triploid individuals that carry two identical sex alleles (one maternal and one paternal) and one non-identical (maternal) sex allele. Would such individuals be viable and if so would they be male or female? Would the *csd* product be functional? Our study shows that Capensis provides an extraordinary experimental system in which to examine the underlying genetics of haplodiploidy and sex determination in bees.
